# Posttraumatic stress disorder correlates among internally displaced Yazidi population following Islamic state of Iraq and Syria attacks in Iraq

**DOI:** 10.1186/s12888-021-03299-8

**Published:** 2021-06-03

**Authors:** Perjan Hashim Taha, Nezar Ismet Taib, Hushyar Musa Sulaiman

**Affiliations:** 1 Psychiatry Unit, College of Medicine, University of Duhok, Nakhoshkhana Road 9, 1014, AM, Duhok, Kurdistan region 42001 Iraq; 2 Duhok Directorate General of Health (DGoH), Duhok, Iraq

**Keywords:** Posttraumatic stress disorder, Internally displaced persons, Iraq, Correlates

## Abstract

**Background:**

In 2014, the so-called Islamic State of Iraq and Syria (ISIS) took over one-third of Iraq. This study measured the rate of posttraumatic stress disorder (PTSD) among Iraqi Yazidi internally displaced persons (IDPs) and examined associated demographic and traumatic risk factors.

**Methods:**

A cross-sectional survey was carried out in April–June 2015 at the Khanke camp, northern Iraq. Trauma exposure and PTSD were measured by the Harvard Trauma Questionnaire (Iraqi version).

**Results:**

Of 814 adult Yazidi IDPs, 34% screened positive for PTSD. Avoidance and intrusion symptoms had the highest means (M = 3.16, SD = 0.86 and M = 2.63, SD = 0.59 respectively). Associated factors of PTSD included exposure to a high number of traumatic events, unmet basic needs and having witnessed the destruction of residential or religious areas (OR = 1.39, 95% CI: 1.02–1.9 and OR = 1.25, 95% CI: 1.01–1.53 respectively). Being a widow was the only linked demographic factor (OR = 15.39, 95% CI: 3.02–78.39).

**Conclusions:**

High traumatic exposure, specifically unmet basic needs and having witnessed destruction, was an important predictor of PTSD among Yazidi IDPs. These findings are important for mental health planning for IDPs in camps.

## Background

At the beginning of 2014, the so-called Islamic State of Iraq and Syria (ISIS) took over large areas of northern and central Iraq, and they controlled one-third of the territory of Iraq by the end of 2014 [1]. The continuous armed conflict in the country led to the displacement of thousands of Iraqi people. The United Nations (UN) estimated that 3.2 million Iraqis were internally displaced persons (IDPs) by the end of 2015 [2]. On August 3, 2014, ISIS launched a very large attack against Yazidi and gained control of the city of Sinjar and the surrounding villages in northwestern Iraq [3]. Almost 6800 people were abducted by ISIS, and approximately half of them were later rescued. Approximately 200,000 people were displaced within a few days, and the majority of them were Yazidis. Yazidis are a religious minority who speak Kurdish and inhabit northern Iraq, Iran and Syria and southeastern Turkey [3]. They refuse religious conversion, which is why they have faced many genocides in their history. The bulk of the displaced people resettled in refugee camps around the city of Duhok in northern Iraq [4].

Refugees and IDPs are a population that is highly vulnerable to mental disorders compared to native and other migrant populations [5]. Exposure to traumatic events in the homeland, such as persecution; torture; combat; imprisonment; threats because of ethnicity, race, or religion; lack of food, water and shelter; separation; and violence to or death of family members, is a common reason for displacement [6]. Posttraumatic stress disorder (PTSD) and depression are the most commonly reported mental disorders with a wide range of occurrence rates [7].

PTSD can be defined as a mental disorder that can occur if an individual experiences, witnesses, or confronts a traumatic event. Patients with PTSD may suffer from intrusive thoughts about the traumatic experience and flashbacks as well as avoidance behavior and hyperarousal symptoms such as irritability and disruptions in sleep and concentration [8]. PTSD is highly prevalent among refugees, immigrants and asylum seekers at a rate of 9–36% compared with the rate of 1–2% in the general population [9]. In addition to severe traumatic events and lack of social support during such situations, the common predictors of PTSD among traumatized individuals are being young, female, having lower socioeconomic status, a family history of psychiatric disorders, and being of a minority race [10, 11]. The number and types of experienced traumatic events are associated with different rates of PTSD [12].

Although many studies have investigated the psychosocial consequences of mass traumas and displacement in different population samples, we aimed to investigate the mental health impact of mass displacement in a population with a long history of multiple genocides added to decades of exposure to internal and external conflicts and political instability. Our study highlights the necessity of mental health assessment, care, and follow-up among this specific population group because although refugee camps provide safe accommodations, displaced people need physical and mental health care. We examined the prevalence of PTSD and investigated the possible correlated factors of PTSD among Yazidi IDPs resettled in refugee camps in Duhok, Iraqi Kurdistan.

## Methods

### Study design and participants

This is a cross-sectional study performed in April–June 2015 as part of a larger community cross-sectional survey among Iraqi IDPs and Syrian refugees resettled in refugee camps in Duhok Province. This research was supported by the Directorate of Health and the Italian nongovernmental organization Association for Solidarity among People (AISPO).

The participants were adults of both genders living in the Khanke camp, which is located 20 km west of the city of Duhok, northern Iraq. In 2015, the number of IDPs in Duhok Province was 483,068, constituting 92,024 families [13]. They were living in the camps and in unfinished structures and school buildings. At the time of the study, the camp population comprised 16,460 individuals living in 3120 tents, most of whom were Yazidi IDPs from Sinjar [14].

The inclusion criterion was that the individual should have a history of displacement following the 2014 ISIS attacks in Iraq. Individuals suffering from mental disorders that affect insight, disturb normal communication or deteriorate cognitive abilities, such as intellectual disabilities, dementia or psychotic disorders, were excluded.

The tent numbers were entered into an Excel sheet, and 814 tents were randomly selected. From each selected tent, one eligible adult fitting the sample inclusion criterion was asked to participate voluntarily. The participant was randomly selected by putting eligible family members’ names on small pieces of paper in a bag and choosing one blindly.

The scientific committee of the College of Medicine/University of Duhok and the Research Ethics Committee of the Duhok Directorate of Health approved the study. The participants provided written informed consents before we conducted the interviews. The interviewer assured the participants of the confidentiality of the gathered data. Of the 814 Yazidi IDPs selected to participate in the current study, only 8 did not consent to participate in the study; in those cases, other family members were randomly selected and asked to participate in the study. A psychiatrist familiar with training in such instruments trained six counselors in the use of the study tools full time for 5 days. The project supervisor, his assistant, and the 6 interviewers were hired locally. The face-to-face interviews lasted for 2 months, beginning on April 15, 2015. The interviews were carried out inside the tents the average length of conducting each of them was approximately 50–60 min.

### Measures

The demographic questionnaire requested the age, gender, marital status, education level, work status, number of siblings, past psychiatric history, and past family history of psychiatric disorders.

The Harvard Trauma Questionnaire (HTQ) is a widely used simple and reliable checklist that measures traumatic experiences, torture exposure, and symptoms [15]. It is beneficial in the assessment of the severity and types of premigration or displacement traumas suffered by survivors of mass violence and common trauma-related psychiatric disorders, such as depression, PTSD and anxiety [16]. Parts I, II and IV of the Iraqi version of the HTQ were administered to the Yazidi IDPs who were fluent in both Arabic and Kurdish languages [17]. Part I comprises 43 items assessing the traumatic events experienced and witnessed [18]. The first 16 items of part IV assess PTSD symptoms [19]. For each item, the individual selects from a 4-point severity scale: “not at all”, “a little”, “quite a bit”, and “extremely”. In the present study, HTQ was used as an interview tool not as a self-assessment questionnaire, because there was a high rate of illiteracy among the IDP population which make it difficult to use a self-reporting method of gathering data. In the present study, the HTQ symptom scale received a Cronbach’s alpha of 0.799, which indicates good internal consistency. If the individual’s HTQ symptom score is ≥2.5, it indicates a likelihood of clinical PTSD.

### Statistical analysis

The gathered data were analyzed using SPSS (software statistical computer package version 22). The preliminary descriptive analysis used frequency tables, including means (M) and standard deviations (SDs), for quantitative data, and percentages were used for qualitative data. The categorical data were tested by chi square. Principal component analysis (PCA) with oblique rotation (δ = 0) was applied to variables of the HTQ part I to designate component groups of traumatic events. The oblique rotation was carried out because we anticipated that the events were correlated with one another. The items with a primary loading greater than 0.30 on the same component were combined. Logistic regression analysis was used to probe the contribution of types of experienced traumatic event and demographic factors to participants’ PTSD symptom levels. Significance was assumed at *P* values < 0.05, and high significance at *P* < 0.001.

## Results

Table 1 demonstrates the sociodemographic characteristics and mental disturbances of the participants at the time of the study. The mean age was 33.71 (SD = 12.84) years, and the range was 77 (18–95) years, but most of the participants were young (18–40 years). There were slightly more males than females (56.4% vs 43.6%). Most of them were married (77.6%). A total of 361 (44.3%) of the participants were illiterate, 33% had completed primary school, and a few had completed high school or held a higher academic degree. The majority were unemployed (*n* = 687, 86.2%). Most of the participants were from large families, and the mean number of siblings was 7.23 (SD = 3.26).

**Table 1:** Sociodemographic characteristics of participants and their PTSD prevalence rates. (*N* = 814). PTSD, PTSD- first 16 questions of HTQ part IV

Sociodemographic characteristics		N	% of total
Ages	18–40 years old	612	75.2
	41–64 years old	164	20.1
	65 years old or more	38	4.7
Gender	Female	355	43.6
	Male	459	56.4
Marital status	Single	159	20
	Married	617	77.6
	Separated	3	0.4
	Widow	16	2
Education level	Illiterate	361	44.3
	Primary	269	33
	Secondary	160	19.7
	Academic	24	2.9
Work status	Employed	110	13.8
	Not employed	687	86.2
Past psychiatric history	Positive	32	3.9
Past family psychiatric history	Positive	53	6.5
Number of siblings	Range, M (SD)	21, 7.23 (3.26)	
**PTSD symptoms**			
PTSD 16 items (Range = 1–4)	M (SD)	2.27 (0.511)	
Intrusion (Range = 1–4)	M (SD)	2.63 (0.598)	
Avoidance (Range = 1–4)	M (SD)	3.16 (0.864)	
Numbing (Range = 1–4)	M (SD)	1.77 (0.650)	
Hyperarousal (Range = 1–4)	M (SD)	2.11 (0.699)	

Only 3.9% had a positive past psychiatric history, and 6.5% had a family history of psychiatric disorders. The PTSD scores measured on the HTQ are also shown in Table 1. PTSD had a mean score of 2.27 (SD = 0.51, R = 1–4). Avoidance and intrusion symptoms had the highest means (M = 3.16, SD = 0.86, R = 1–4 and M = 2.63, SD = 0.59, R = 1–4, respectively) compared to numbing and hyperarousal symptoms.

Table 2 displays typing and subtyping of common traumatic events, item loading, and frequencies and percentages of experiencing or witnessing among the participants. Prior to the PCA step, the frequencies and percentages of exposure to traumatic events were studied. Traumatic events that were extremely rare (experienced by less than 5%) were deleted because they did not provide enough information to maintain a meaningful grouping of items. From a total of 48 items, 25 were deleted. Examples of uncommon traumatic events were sexual violence; brainwashing; forced labor; witnessing the torture, murder, arrest or execution of others; witnessing chemical attacks; being confined to home; being forced to pay for bullets used to kill family members; receiving the body of a family member and being prohibited from mourning and burial rites; and having someone inform against a participant. Another item (suffering ill health without access to medical care) was also canceled because its primary loading was less than 0.30 on the same factor. As a result, 21 variables remained that were suitable for PCA.

**Table 2:** Typing and Subtypes of traumatic events, item loading, and frequencies and percentages of occurring among IDPs. (*N* = 814)

Traumatic event	1	2	3	4	5	6	N (%)
Tortured (physical or mental suffering)	0.79						90(11.1)
Searched	0.71						150(18.5)
Oppressed because of ethnicity, religion, or sect	0.67						514(63.3)
Exposed to combat situation	0.47						388(47.8)
Forced to change religion	0.45						335(41.4)
Disappearance, hostage or kidnapping of family member		0.68					109(13.4)
Murder or violent death of friend		0.64					216(26.7)
Murder or violent death of family member		0.57					112(13.8)
Disappearance, hostage, or kidnapping of friend		0.55					316(39)
Serious physical injury of family member or friend		0.45					64(7.9)
Witnessed video film on a known person or places, content is violence		0.33					49(6)
Forced to leave hometown			0.91				774(95.3)
Expelled from city based on ancestral origin, religion, or sect			0.89				764(94.1)
Lack of food or clean water				0.82			635(78.1)
Lacked shelter				0.81			741(91.3)
Witnessed desecration or destruction of religious shrines					0.76		124(15.4)
Witnessed shelling, burning, or razing of residential areas					0.76		102(12.7)
Witnessed rotting corpses					0.57		126(15.6)
Forced evacuation under dangerous conditions						0.71	765(94)
Forced to hide						0.60	695(85.6)
Confiscation, looting, or destruction of personal property						0.57	785(96.4)

Types of traumatic events: 1. Trauma or persecution to self, 2. Trauma or abduction of others, 3. Forced immigration, 4. Lack of basic necessities, 5. Witnessed destruction, and 6. Coercion.

PCA yielded 6 trauma components, which altogether produced a cumulative variance of 50.1%. Every traumatic event subtype had a loading greater than 0.30 on one component and did not have a loading greater than 0.30 on the next component. The traumatic events were sorted as follows: 1. Trauma to or persecution of self, 2. Trauma to or abduction of family member or friend, 3. Forced immigration, 4. Lack of basic necessities, 5. Witnessed destruction, and 6. Coercion.

Analysing the results of Part II of HTQ revealed that 335 (41.1%) of Yazidi IDPs reported that the most terrifying event experienced was witnessed the killing of people (including family members, relatives or friends) by the ISIS solders during their attacks over the city of Sinjar and the surrounding villages at the first few days of the attack (3rd -4th of August, 2014). Nearly half of participants 405 (49.7%) reported evacuation under dangerous conditions. The remaining 9.2% reported other subtypes of traumatic events.

**Fig. 1:**
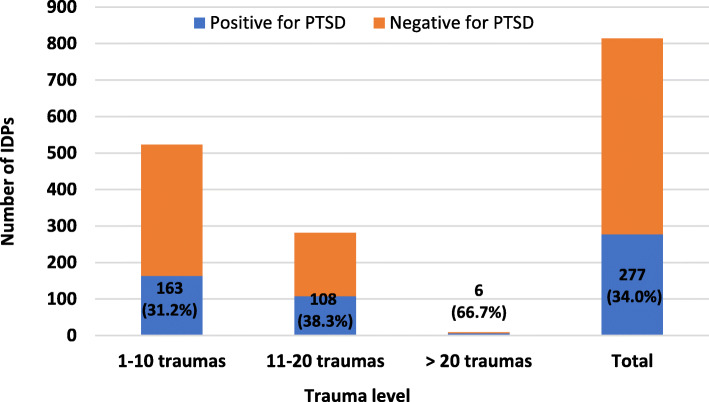
Cumulative trauma events and rate of PTSD among IDPs. (*N* = 814)

Figure 1 shows the cumulative trauma events and rates of PTSD among the participants. The prevalence of PTSD among them according to the cutoff scores was 34%. Among those who were mildly traumatized (1–10 traumatic events), only 163 (31.2%) screened positive for PTSD compared to 68.8% who screened negative. Only 108 (38.3%) of the IDPs who experienced 11–20 traumatic events screened positive for PTSD. Of those who were severely traumatized (> 20 traumatic events), 6 (66.7%) had a diagnosis of PTSD compared to only 3 (33.3%) who did not. All these differences were statistically significant (X^2^ = 8.469, *P* = 0.014).

Logistic regression analysis of subtypes of experienced traumatic event components as predictors of PTSD is demonstrated in Table 3. Among the 6 types of traumatic components yielded by the PCA method, the types of individual trauma exposure that were significant predictors of the development of PTSD were unmet basic needs and having witnessed destruction (OR = 1.393, *P* = 0.037, 95% CI for OR = 1.021–1.901 and OR = 1.25, *P* = 0.033, 95% CI for OR = 1.018–1.534, respectively). Lower PTSD scores were associated with forced migration (OR = 0.552, *P* = 0.003, 95% CI for OR = 0.373–0.815).

**Table 3:** Traumatic events (by HTQ) and sociodemographic variables as predictors of PTSD among Yazidi IDPs. (*N* = 814)

Predictors		***P***-value	OR	95%CI for OR
**Traumatic events**				
Trauma or persecution to self		0.461	1.081	0.879–1.329
Trauma or abduction to others		0.532	0.956	0.830–1.101
Forced immigration		0.003*	0.552	0.373–0.815
Lack of basic needs		0.037*	1.393	1.021–1.901
Witnessed destruction		0.033*	1.250	1.018–1.534
Coercion		0.553	0.908	0.660–1.249
**Sociodemographic factors**	**(reference)**			
Age		0.322	1.007	0.993–1.022
Gender	Male (ref)		1.00	
	Female	0.120	0.749	0.520–1.079
Marital status	Single (ref)		1.00	
	Married	0.738	1.092	0.651–1.833
	Separated	0.203	5.052	0.417–61.235
	Widow	0.001*	15.391	3.022–78.396
Education	Illiterate (ref)		1.00	
	Primary	0.303	0.816	0.555–1.201
	Secondary	0.794	0.933	0.556–1.567
	Academic	0.506	0.704	0.250–1.981
Work status	Employed (ref)		1.00	
	Unemployed	0.519	1.172	0.724–1.899
Number of siblings		0.056	1.050	0.999–1.104
Psychiatric history	Negative (ref)		1.00	
	Positive	0.878	0.935	0.396–2.208
Family history	Negative (ref)		1.00	
	Positive	0.307	0.700	0.353–1.387

*HTQ* Harvard trauma questionnaire, *PTSD* PTSD- first 16 questions of HTQ part IV, *OR* Odd ratio, *CI* Confidence interval, *P-value < 0.05

To study the sociodemographic correlates (age, gender, marital status, education level, occupation, and number of siblings) for PTSD, a binary logistic regression model was also used (Table 3). The table shows that sociodemographic variables were not risk factors for developing PTSD among the Yazidi IDPs except that being a widow was a strongly significant predictor (OR = 15.391, *P* = 0.001, 95% CI for OR = 3.022–78.396).

## Discussion

Our study provides evidence of mental distress after exposure to traumatic events endured by Yazidi IDPs resettled in IDP camps following the violent attacks by ISIS in 2014. It showed a 34% prevalence rate of PTSD. A higher number of traumatic events was associated with a higher rate of PTSD. Predictive traumatic events for PTSD were unmet basic needs and having witnessed destruction. Demographic factors did not predict PTSD except that being widowed increased the rate of PTSD by 15 times.

The high prevalence rate of PTSD (34%) found in our study is consistent with the rates of PTSD revealed in other studies performed on Iraqi refugees and displaced people after the events of 2014. A systematic review of the literature describing the prevalence rates of PTSD among resettled Iraqi refugees in Western countries finds a range from 8 to 37.2% [20]. Asylum seekers, refugees, and IDPs displaced to Iraqi Kurdistan showed a PTSD prevalence rate of 48.7% [21]. Tekin et al. [22] showed a rate of 42.9% among displaced Iraqi Yazidis. The rate was 36.4% among Yazidi children and adolescents who had immigrated to Turkey from Iraq [23]. Among other refugees inhibiting Iraqi Kurdistan camps, such as Kurdish Syrian refugees, the estimated levels of PTSD symptoms ranged between 35 and 38% [24]. This finding indicates the impact of mass conflict and displacement and the severity of the traumatic events that these respondents had experienced, including a combination of war and political, religious and ethnic violence. Several factors, such as different methodological approaches and demographic characteristics, may contribute to the differences in PTSD rates reported in other studies.

Among the 4 categories of specific PTSD symptoms, the avoidance symptoms suffered by the Yazidi IDPs participating in our study showed the highest means. Symptoms of intrusion were also relevant, with high means compared to numbing and hyperarousal symptoms. Iraqi refugees in Germany presented similarly, with nightmares as the most prominent symptom of PTSD [25].

Traumatic events among refugees and IDPs are not presented individually, but they are usually accumulated and interconnected [23]. There is a positive correlation between the number of experienced traumatic events and vulnerability to developing mental disorders [6]. Our study presented the effect of the number of traumatic events on the rate of PTSD. Approximately 66.7% of those who had experienced more than 20 traumatic events developed symptoms of PTSD. This indicates the cumulative effect of exposure to multiple traumatic events before and during the displacement time. The cumulative trauma index accounts for the increased prevalence rate of PTSD among Iraqi refugees [19]. This finding supports the dose-response relationship between traumatic exposure and PTSD among refugees in post-conflict periods [26]. Among Syrian refugees in Turkey, experiencing 2 or more traumatic events was a significant predictor of PTSD [27].

Different subtypes of experienced traumatic events had different effects as predictors of PTSD. There were significant positive correlations between traumatic events and PTSD symptoms among Kurdish Syrian refugees in Iraqi Kurdistan camps [24]. In our study, the most significant predictors for the development of PTSD were unmet basic needs and having witnessed destruction. Unmet basic needs included becoming homeless with no access to health care and lack of food and water. This finding indicates that being deprived of basic elements of survival can have a predictive value in the development of PTSD among IDPs because of its direct impact on individuals. Having witnessed destruction included witnessing the destruction of religious shrines; the shelling, burning, or razing of residential areas; and rotting corpses. Witnessing these violent events, which are associated with losing the homeland and religious persecution, exacerbates war impacts. The most painful or terrifying traumatic event recounted by the survivors of “Anfal”, a military operation against the Kurds of northern Iraq, was witnessing murder [28]. Additionally, trauma subtypes provided even more entropy than the cumulative trauma effect in the prediction of PTSD [19]. The study showed that unmet basic needs was a stronger risk factor for depression than for PTSD.

When we searched for sociodemographic predictors of PTSD among Yazidi IDPs, marital status was the only component that acted as a predictive factor. The severity of experienced traumatic events and the short period between traumatic exposures and the assessment (less than 1 year) made the IDPs perceive the traumas more collectively and more similarly than respondents in other studies. A study conducted on Yazidis found that no sociodemographic predictors for PTSD among the surviving Yazidi women and girls except the number of family members directly affected by ISIS [4]. Being a widow increased the risk of developing PTSD by 15 times. Not having a partner is associated with poor mental health owing to the lack of the social support provided by a partner and the sense of increased responsibility for raising the children and increased worry about the future. Additionally, the widowed women may have witnessed the killing of their husbands by ISIS solders. This finding indicates that poor social support is a strong predictor of PTSD among Yazidi IDPs. Those who were widowed prior to the 2014 events may have been affected by prior loneliness and its mental consequences, aggravating their vulnerability to PTSD [29]. Following traumatic events, the incidence of PTSD is significantly higher in widows than in married women with living spouses. A systemic review of 11 studies on mood and anxiety disorders in widows revealed higher incidence of PTSD during the first year after bereavement compared to control subjects [30].

This study is not devoid of limitations. Firstly, we cannot attribute causation because of the cross-sectional design of the study. Secondly, because the sample was taken from only one IDP camp, omitting IDPs inhabiting informal settlements and within cities, it is difficult to determine whether it is representative. Thirdly, as all the 6 interviewers were males, the female respondents might have difficulty in reporting specific sensitive traumatic events. Fourthly, although those with a history of psychiatric disorders prior to displacement were excluded from participating in the study, we lack adequate information on the trauma experiences of the participants prior to the ISIS attacks that may make it difficult to determine a link between previous trauma experiences and current PTSD symptoms. Lastly, the interviews conducted 6 months after ISIS attacks and previous possible psychotherapy was not assessed which might be helpful in knowing if the recovery may play a role in the investigated associations. The strength of this study is the careful assessment of PTSD by six well-trained Iraqi psychological counselors who did not need the help of interpreters and did not use self-reported questionnaires. Additionally, they were able to select probable cases and refer them to suitable mental health services. Furthermore, this is a rare study that highlights the cumulative and qualitative effects of trauma exposure in IDPs who have recently escaped from severe terroristic attacks to displacement camps.

## Conclusions

The results of this study provide evidence of the high prevalence rate of PTSD among Yazidi IDPs living in displacement camps in northern Iraq following ISIS attacks. The commonest PTSD symptoms among them were avoidance and intrusion symptoms. The number of experienced traumas was positively correlated with the symptom severity of PTSD. Unmet basic needs and having witnessed destruction were the most impactful traumatic events. The results of our study provide a better understanding of the mental health of Yazidi IDPs and a cross-cultural understanding of the effects of mass conflicts and displacement. They may provide an urgent warning and a call for multidisciplinary interventions for this group of people. Furthermore, the results of this article have possible applications for authorities in charge, governmental, nongovernmental organizations, health care providers and those who supply psychosocial support for Yazidi IDPs. In addition to the limited available psychological service resources in the locality, the health care professionals working in displacement camps should be familiar with trauma experiences and should be trained on psychological interventions.

## Data Availability

All data generated, analysed or described in this manuscript, including all relevant raw data, will be freely available to any scientist wishing to use them for non-commercial purposes, without breaching participant confidentiality.

## Abbreviations

ISIS: Islamic State of Iraq and Syria
PTSD: Posttraumatic stress disorder
IDPs: Iraqi internally displaced persons
HTQ: Harvard Trauma Questionnaire
UN: The United Nations
AISPO: Association for Solidarity among People (Italian nongovernmental organization)
SPSS: Statistical Package for the Social Sciences
PCA: Principal component analysis
